# ^18^F-FDG PET/CT-derived total lesion glycolysis predicts abscess formation in patients with surgically confirmed infective endocarditis: Results of a retrospective study at a tertiary center

**DOI:** 10.1007/s12350-023-03285-5

**Published:** 2023-06-01

**Authors:** Sabine Julia Maria Sag, Karin Menhart, Florian Hitzenbichler, Christof Schmid, Frank Hofheinz, Jörg van den Hoff, Lars Siegfried Maier, Dirk Hellwig, Jirka Grosse, Can Martin Sag

**Affiliations:** 1 Department of Internal Medicine II/Cardiology, University Hospital Regensburg, Franz-Josef-Strauß-Allee 11, 93053 Regensburg, Germany; 2 Department of Nuclear Medicine, University Hospital Regensburg, Regensburg, Germany; 3 Department of Infection Prevention and Infectious Diseases, University Hospital Regensburg, Regensburg, Germany; 4 Department of Cardiothoracic Surgery, University Hospital Regensburg, Regensburg, Germany; 5 PET Center, Institute of Radiopharmaceutical Cancer Research, Helmholtz-Zentrum Dresden-Rossendorf, Dresden, Germany

**Keywords:** ^18^F-FDG PET/CT, infective endocarditis, total lesion glycolysis, valve abscess

## Abstract

**Background:**

Abnormal activity of ^18^F-FDG PET/CT is a major Duke criterion in the diagnostic work-up of infective prosthetic valve endocarditis (IE). We hypothesized that *quantitative* lesion assessment by ^18^F-FDG PET/CT-derived standard maximum uptake ratio (SURmax), metabolic volume (MV), and total lesion glycolysis (TLG) might be useful in distinct subgroups of IE patients (e.g. IE-related abscess formation).

**Methods:**

All patients (*n* = 27) hospitalized in our tertiary IE referral medical center from January 2014 to October 2018 with preoperatively performed ^18^F-FDG PET/CT *and* surgically confirmed IE were included into this retrospective analysis.

**Results:**

Patients with surgically confirmed abscess formation (*n* = 10) had significantly increased MV (by ~ fivefold) and TLG (by ~ sevenfold) as compared to patients without abscess (*n* = 17). Receiver operation characteristics (ROC) analyses demonstrated that TLG (calculated as MV × SURmean, i.e. TLG (SUR)) had the most favorable area under the ROC curve (0.841 [CI 0.659 to 1.000]) in predicting IE-related abscess formation. This resulted in a sensitivity of 80% and a specificity of 88% at a cut-off value of 14.14 mL for TLG (SUR).

**Conclusion:**

We suggest that ^18^F-FDG PET/CT-derived quantitative assessment of TLG (SUR) may provide a novel diagnostic tool in predicting endocarditis-associated abscess formation.

**Supplementary Information:**

The online version contains supplementary material available at 10.1007/s12350-023-03285-5.

## Introduction

^18^F-FDG PET/CT imaging has become increasingly important in the diagnostic work-up of infective endocarditis (IE).^1,2^ The 2015 guidelines of the European Society of Cardiology (ESC) define abnormal activity of ^18^F-FDG PET/CT as major criterion of the modified Duke Criteria in the diagnostic work-up of infective endocarditis (IE) in patients with prosthetic valves and consider a pathological distribution of ^18^F-FDG within the suspected lesion as *qualitatively* relevant.^3^ In daily clinical practice, the standard maximum uptake value (SUVmax) of ^18^F-FDG is detected and used to interpret target lesions.^4^ However, interpretation of SUVmax may be challenging due to numerous possible confounders including the patient’s blood glucose level at the time of ^18^F-FDG injection, the waiting time after application until PET acquisition (denoted by *T*), or technical issues like cross-calibration between dose calibrator and PET scanner.^4,5^ The standard uptake ratio (SUR), defined as uptake time-corrected ratio of lesional SUV to aortic blood pool SUV (SUVblood), essentially eliminates most of these shortcomings and leads to an improved correlation with the metabolic uptake rate (derived via Patlak analysis of dynamic PET scans) as compared to SUV alone.^5^ In addition, SUR provides an improved test–retest stability and an increased prognostic value compared to SUV.^6–8^

Beside uptake parameters, like SUV and SUR, used as surrogate measures of glucose consumption, there are further quantitative measures, namely metabolic volume (MV, referred to as metabolic tumor volume in the field of oncology) and total lesion glycolysis (TLG, calculated as the product of MV × SUVmean) that both do take into account the *volumetric extent* of a ^18^F-FDG-positive lesion. In the non-cardiac setting, MV and TLG have shown reliable correlations with serologic markers of tumor burden (e.g. LDH and S-100 protein) and inflammation (e.g. CRP).^5,9^ Moreover, changes of MV and TLG during oncological therapy correlate with treatment response and clinical outcome in a variety of tumor entities.^4,10–14^ However, very little is known with respect to the performance of these quantitative ^18^F-FDG PET/CT-derived parameters in the setting of infection, particularly in IE. We hypothesized that *quantitative* lesion assessment by ^18^F-FDG PET/CT-derived standard maximum uptake ratio (SURmax), metabolic volume (MV), and total lesion glycolysis (TLG) might be useful in distinct subgroups of patients suffering from IE (such as in patients with IE-related abscess formation).

## Methods

This retrospective analysis has been approved by the institutional ethics committee (No. 18-972-104). Requirement for written informed consent was waived for this retrospective analysis. Our study cohort and ^18^F-FDG PET/CT image acquisition protocol were described previously.^15^ The same study population was used in an earlier study.^15^ However, for the current analysis, we only included patients with preoperatively performed ^18^F-FDG PET/CT *and* surgically confirmed IE (*n* = 27). Please note that the number of IE affection sites is higher than the number of patients because some patients had more than one IE affection site (see Table 1).

**Table 1:** Baseline clinical and echocardiographic characteristics

	*n* = 27
Age, years	64 [57–69]
Sex (male)	23 (85)
Diabetes mellitus	3 (11)
Prior history of IE	2 (7)
Intrahospital mortality	8 (30)
Intrahospital IE	3 (11)
Prior admission to other hospital	20 (74)
Echocardiographic findings	
Impairment of LVEF	8 (30)
Time to initial TOE, days	0.0 [− 4.0–2.0]
Time to TOE at tertiary hospital, days	3.0 [1.0–4.5]
Initial TOE negative	1 (4)
Primary Duke criterion positive	25 (93)
Native valve IE	6 (22)
Prosthetic valve IE	17 (63)
Mechanical	4/17
Biological	9/17
Reconstruction	4/17
Including replacement of ascending aorta	2/17
Time since implantation, years	4.0 [0.5–8.0]
Valves implanted > 1 year	12/17
Valves implanted < 3 months	4/17
Valves implanted 3–12 months	1/17
Cardiac device-related IE	7 (26)
Cardiac device-related IE without concomitant affection of a cardiac valve	4/7
Pacemaker	3/7
ICD	2/7
CRT-D	2/7
Time since implantation, years	2.5 [1.3–6.8]
Device implanted > 1 year	6/7
Device implanted < 3 months	1/7
Device implanted 3–12 months	0/7
Vegetation	22 (82)
IE affection site	
Aortic	15 (56)
Mitral	10 (37)
Pulmonary	1 (4)
Tricuspid	3 (11)
Cardiac device	7 (26)
Vegetation size, overall, mm	18 [12–23]
NPVE, mm	20 [11–26]
PVE, mm	17 [11–22]
Abscess	
Definite	4 (15)
Inconclusive	5 (19)
Fistula	0 (0)
Prosthetic valve dehiscence	1/18
Paravalvular leakage	3/18

Values represent the median [interquartile range] or numbers (percentages)

*CRT-D* cardiac resynchronisation therapy defibrillator, *ICD* implantable cardioverter defibrillator, *IE* infective endocarditis; *LVEF* left ventricular ejection fraction, *NPVE* non-prosthetic valve endocarditis, *PVE* prosthetic valve endocarditis, *TOE* transesophageal echocardiography

### Patient preparation and PET/CT image acquisition protocol

Patients were scanned on a Biograph 16 PET/CT scanner (CTI-Siemens, Erlangen, Germany) consisting of a 16-slice multidetector CT (0.5 seconds per revolution) and on a Biograph mCT 40 FLOW PET/CT scanner (CTI-Siemens, Erlangen, Germany), consisting of a 40-slice multidetector CT (0.5 seconds per revolution). After a fasting period of at least 6 hours, 3-MBq ^18^F-FDG per kilogram body weight were injected intravenously (256 ± 43 MBq). The patients’ blood glucose level was strictly controlled to be below 150 mg/dL (8.32 mmol/L). To increase renal tracer elimination, patients received an injection of 20 mg furosemide as well as intravenous hydration shortly after ^18^F-FDG injection. Heparin was not routinely used. In order to minimize muscular ^18^F-FDG uptake, patients were advised to stay in a quiet lying position. Warming blankets were used to avoid freezing of the patients and to keep potential tracer accumulation in brown fat tissue to a minimum. Patients were instructed to void the bladder prior to scanning and to remove all metal parts. It needs to be mentioned that there is controversy with respect to the ideal point in time at which image acquisition should be performed following tracer injection, which is of particular relevance for distinctly uptake time-dependent parameters, such as SUV (but much less so or not at all for SUR). Our protocol used standard acquisition times following tracer injection (i.e. ~ 60 minutes post injection), which were previously shown to be associated with higher specificity rates as compared to later acquisition times (i.e. ~ 150 minutes post injection) in patients with PVE.^16^ Patients included in our study were scanned at a median time of 67 minutes (IQR 56 to 91 minutes) post tracer injection and should therefore be within the optimal time frame for image acquisition. Using the Biograph 16 PET/CT scanner, images of the trunk were acquired with elevated arms (pelvis to skull or skull base). Depending on the patient size and clinical indication, six to eight overlapping bed positions with three minutes of PET acquisition time each were used. Using the Biograph mCT 40 FLOW PET/CT scanner, images of the whole body (skull to feet) were acquired using the continuous bed move (torso: 0.8 cm/min, legs: 1.1 cm/min). The same area was covered by a low-dose CT scan (tube current 50 mAs, tube voltage 120 kV). No contrast agents were given. PET images (slice thickness 5 mm) were corrected for random coincidences, decay, scatter, and attenuation and reconstructed iteratively using the ordered subsets expectation maximization algorithm (OSEM) with four iterations and eight subsets. PET images were scaled to allow SUV measurements. PET and CT images were checked for breathing artifacts. Only PET images without ECG gating were used for reanalysis.

### Image interpretation

^18^F-FDG PET/CT images were reanalyzed by two independent nuclear medicine physicians blinded to patients’ characteristics using syngo.via version VB40 (Siemens Healthineers, Germany). Attenuation corrected as well as uncorrected images were inspected. Abnormal focal or diffuse ^18^F-FDG uptake (without using a fixed threshold, see below) as compared to surrounding blood pool, corresponding to cardiac valve, prostheses, or intracardiac devices (lead and/or pocket) was considered positive for IE. According to the PET/CT fusion image, a volume of interest (VOI) was outlined within the infection site using ROVER version 3.0.35 (ABX, Radeberg, Germany) and syngo.via version VB40 (Siemens Healthineers, Germany). Care was taken to ensure that possible adjacent foci unrelated to inflammation were not included in the VOI. SUVmax and SUVmean of cardiac inflammatory lesions were measured and calculated automatically. To determine the SUVmean of the liver parenchyma, a spherical VOI with a diameter of at least 3 cm was placed in normal liver parenchyma of the right lobe (volume ≥ 14.1 mL), excluding visible vessels and potential liver lesions. A thread-like VOI in the aorta was automatically generated after setting a seed point in the proximal part of the descending thoracic aorta (volume about 8 mL). SUVmean of the aortic blood pool (SUVblood) was determined by semiautomatic delineation of the aorta in the attenuation CT. The resulting intraluminal VOI was then automatically transferred to the co-registered PET image, and the mean value was set as the blood pool value. SURmax was computed as ratio of SUVmax to SUVblood. Uptake-time correction was performed to *T*0 = 75 minutes as described in^5,17^: SURmax = (*T*0/*T*) × (SUVmax/SUVblood), where *T* is the actual time of measurement in the respective scan.

To quantify MV, a delineation threshold is calculated using the following formula according to^18^: SUVmean (liver VOI) + 2.5 standard deviation. Lesions with a SUVmax below this threshold were considered *negative*. For delineation of suspected lesions, a relative threshold of 41% of the SUVmax was used. The 41% threshold is motivated by the fact that this value corresponds well with the real extend of an FDG accumulation with irregular shape as shown by phantom experiments to validate the measurement of metabolic tumor volume in lymphoma.^19^

Lesion TLG (SUV) and TLG (SUR) were calculated as the respective product of mean uptake with MV:

- TLG (SUV) = SUVmean x MV,
- TLG (SUR) = SURmean x MV.

### Statistical analysis

Data were analyzed using the SPSS statistical software package (SPSS 26.0, IBM SPSS Statistics, Armonk, New York, USA). Descriptive statistics are presented as median and interquartile range (IQR) for continuous data and as number and percentages for categorical data. Mann–Whitney *U* Test was used to compare median values for independent data. Categorical parameters were evaluated by Chi-squared test. Linear regression models were calculated to assess the association of MV, TLG (SUV), TLG (SUR), SURmax, and SUVmax with CRP and leukocytes count. Receiver operating characteristic (ROC) analysis was performed for determination of threshold values and for calculation of sensitivities and specificities. All reported *P*-values are two-sided, with .05 considered the threshold for statistical significance.

## Results

### Clinical characteristics of study population

Baseline clinical and echocardiographic characteristics of 27 patients with surgically confirmed IE and preoperatively performed ^18^F-FDG PET/CT are displayed in Table 1. Median age was 64 years (IQR: 57 to 69 years) and the majority of patients were male (85%). Eight patients (30%) died during hospital stay. Six patients (22%) had native valve IE (NVE), 17 patients (63%) had prosthetic valve IE (PVE), and four patients (15%) had isolated cardiac device-related IE (CDRIE). Vegetations were identified in 22 patients (82%). All patients underwent echocardiography. Endocarditis-associated abscess formation was diagnosed in four patients (15%) by transesophageal echocardiography (TOE), while no abscess was detected using transthoracic echocardiography (TTE). The main IE affection site was aortic valve region. Laboratory values and microbiological findings (Table 2) demonstrate that median CRP was 148.7 mg/L (IQR: 66.7 to 281.3 mg/L) at the time of admission. Twenty-five patients (93%) had positive blood cultures and the most frequent causative pathogen was Staphylococcus aureus (60%). Table 3 depicts technical aspects and findings using ^18^F-FDG PET/CT. Time between first positive blood culture and ^18^F-FDG PET/CT was 8 days (IQR: 5 to 15 days). CRP at the time of PET scan was 88.6 mg/L (IQR: 42.9 to 121.0 mg/L) and leukocytes were 10.8/nL (IQR: 7.6 to 12.9/nL). At that point in time, 24 patients (89%) received antibiotic therapy. Median vegetation size at the time of PET scan was 14 mm (IQR: 9 to 20 mm). Fasting glucose level at the time of ^18^F-FDG injection was 101 mg/dL [IQR: 89 to 139 mg/dL], the waiting time after ^18^F-FDG application until PET acquisition was 67 minutes [IQR: 56 to 91 minutes], and SUV_blood_ was 1.8 [IQR: 1.5 to 1.9]. SURmax ranged from 1.0 to 5.3. Table 4 shows results of modified Duke Classifications at admission, at time of ^18^F-FDG PET/CT acquisition, and at the end of hospital stay. Surgery confirmed endocarditis-associated abscess formation in 10 out of 27 patients (37%).

**Table 2:** Laboratory values and microbiologic findings

	*n* = 27
CRP at the time of admission, mg/L	148.7 [66.7–281.3]
Leukocytes count at the time of admission, /nL	11.8 [9.1–15.5]
PCT at the time of admission, ng/mL	1.37 [1.07–20.85]
Blood cultures available	27 (100)
Blood cultures positive	25 (93)
Primary Duke criterion positive	22 (82)
Causative pathogen	
*Staphylococcus aureus*	15/25
*Enterococci*	1/25
Coagulase-negative *staphylococci*	4/25
*Streptococci*	3/25
HACEK	1/25
*Candida* *subspecies*	1/25
Antibiotic therapy before blood cultures	5 (19)
Empiric antibiotic therapy	24 (89)

Values represent the median [interquartile range] or numbers (percentages)

*CRP* C-reactive protein, *PCT* procalcitonin;

**Table 3:** Technical aspects and findings using ^18^F-FDG PET/CT

	*n* = 27
Time to ^18^F-FDG PET/CT since external admission, days	10 [7–20]
Time to ^118^F-FDG PET/CT since admission at tertiary center, days	4 [3–8]
Time between ^18^F-FDG PET/CT and surgery, days	9 [4–14]
Indication for ^18^F-FDG PET/CT	
Inconclusive echocardiography	5 (19)
Other foci/septic emboli	10 (37)
Combination of both	12 (44)
Time from first positive blood culture to ^18^F-FDG PET/CT, days	8 [5-15]
Duration of antibiotic therapy before ^18^F-FDG PET/CT, days	2 [− 1–7]
CRP at the time of ^18^F-FDG PET/CT, mg/L	88.6 [42.9–121.0]
Leukocytes count at the time of ^18^F-FDG PET/CT, /nL	10.8 [7.6–12.9]
Fasting glucose, mg/dL	101 [89–139]
Fever at the time of ^18^F-FDG PET/CT	3 (11)
^18^F-FDG PET/CT during antibiotic therapy	24 (89)
Pathogen-directed therapy	21 (78)
Vegetation size at the time of ^18^F-FDG PET/CT, mm	14 [9-20]
Inadequate myocardial suppression	8 (30)
Time from ^18^F-FDG injection to PET acquisition, min	67 [56–91]
^18^F-FDG PET/CT result inconclusive	0 (0)
^18^F-FDG PET/CT positive	15/27
True positive	15/15
False positive	0/15
^18^F-FDG PET/CT negative	12/27
True negative	0/12
False negative	12/12
SUVmax	4.9 [3.6–6.2]
SURmax	2.5 [1.8–3.0]
TLG (SUV), mL	49.1 [29.6–73.7]
TLG (SUR), mL	6.2 [1.9–17.8]
MV, mL	20.9 [11.4–28.0]
Septic emboli detected by ^18^F-FDG PET/CT	4 (15)

Values represent the median [interquartile range] or numbers (percentages)

*MV* metabolic volume, *SURmax* standard maximum uptake ratio, *SUVmax* standard maximum uptake value, *TLG* total lesion glycolysis

**Table 4:** IE classification according to modified Duke criteria and definite surgical findings

	*n* = 27
Modified Duke classification at admission	26 (96)
Definite IE	15/27
Possible IE	7/27
Rejected	4/27
Modified Duke classification at the time of ^18^F-FDG PET/CT	27 (100)
Definite IE	21/27
Possible IE	5/27
Rejected	1/27
Modified Duke classification at the end of hospital stay, *n* (%)	27 (100)
Definite IE	22/27
Possible IE	5/27
Rejected	0/27
Definite IE (microbiological, histopathological or surgical confirmation)	
Microbiology available	24 (89)
Microbiological confirmation of IE	12/24
Culture positive	4/12
PCR positive	11/12
Histopathology available	12 (44)
Histopathological confirmation of IE	9/12
Detection of germs	5/9
Surgical confirmation of IE	27 (100)
Intraoperative abscess	10/27

*IE* infective endocarditis, *PCR* polymerase chain reaction

### No correlation of quantitative ^18^F-FDG-derived PET parameters with markers of systemic inflammation

Linear regression analysis failed to show a significant association of SUVmax, SURmax, MV, TLG (SUV), or TLG (SUR) with CRP or with leukocytes count. Furthermore, there was no significant correlation of SUVmax, SURmax, MV, TLG (SUV), or TLG (SUR) with overall vegetation size nor with vegetation size at the time of ^18^F-FDG PET/CT (data not shown).

### Performance of quantitative ^18^F-FDG-derived PET parameters in distinct IE subgroups

**Figure 1:**
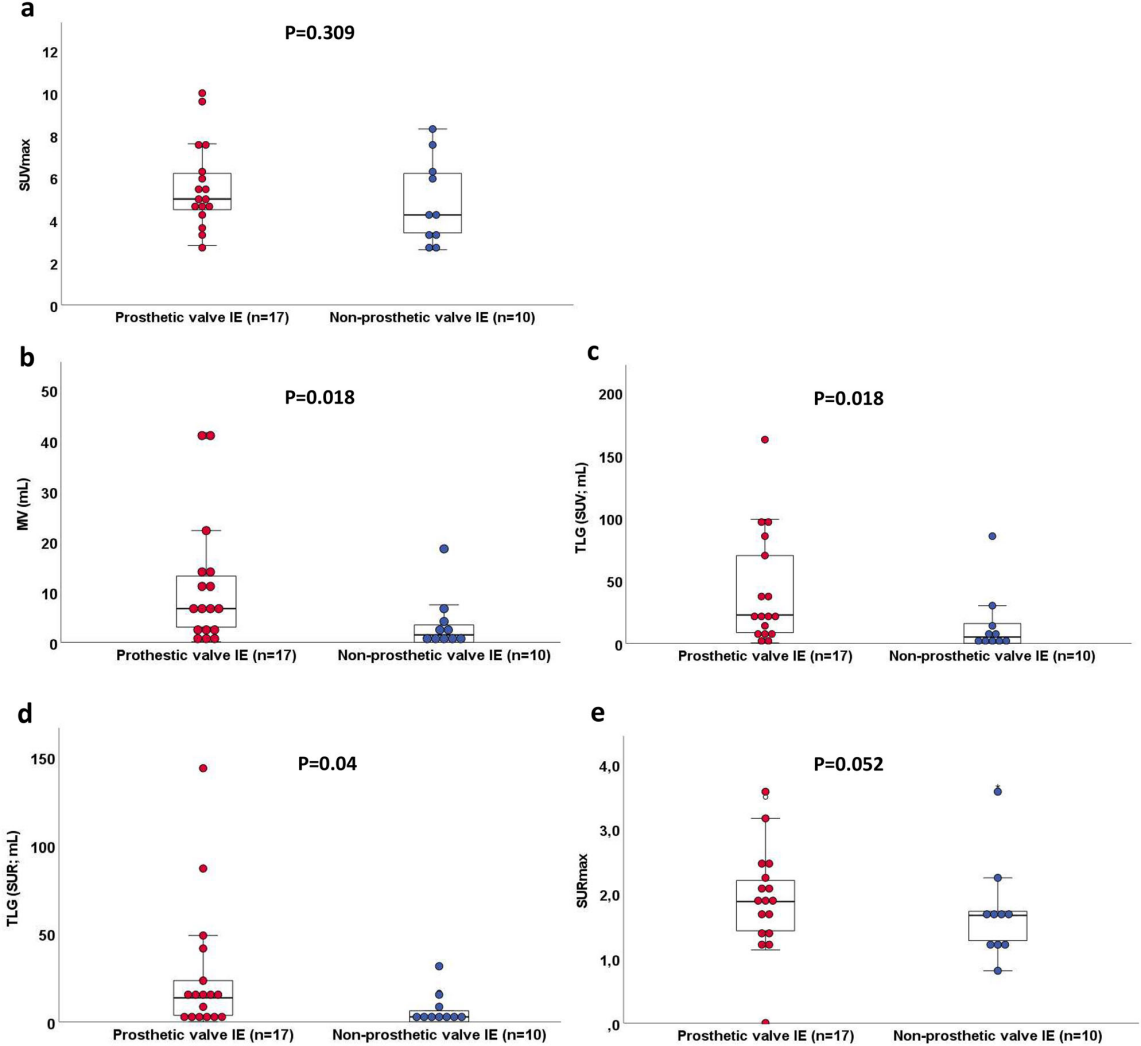
Performance of quantitative ^18^F-FDG PET/CT-derived PET parameters in PVE vs. NPVE. Box and dot plots of **a** SUVmax, **b** MV, **c** TLG (SUV), **d** TLG (SUR), and **e** SURmax according to the type of IE (prosthetic valve vs. non-prosthetic valve IE) comparing the median of according values. Box and whisper plots represent the median and the 25, 75, 10, and 90% quantiles, and dots represent individual data points. Significantly higher values for MV, TLG (SUV), and TLG (SUR) were observed in patients with PVE (red) as compared to patients with NPVE (blue). *IE* infective endocarditis, *MV* metabolic volume, *NPVE* non-prosthetic valve endocarditis, *PVE* prosthetic valve endocarditis, *SURmax* standard maximum uptake ratio, *SUVmax* standard maximum uptake value, *TLG* total lesion glycolysis

**Figure 2:**
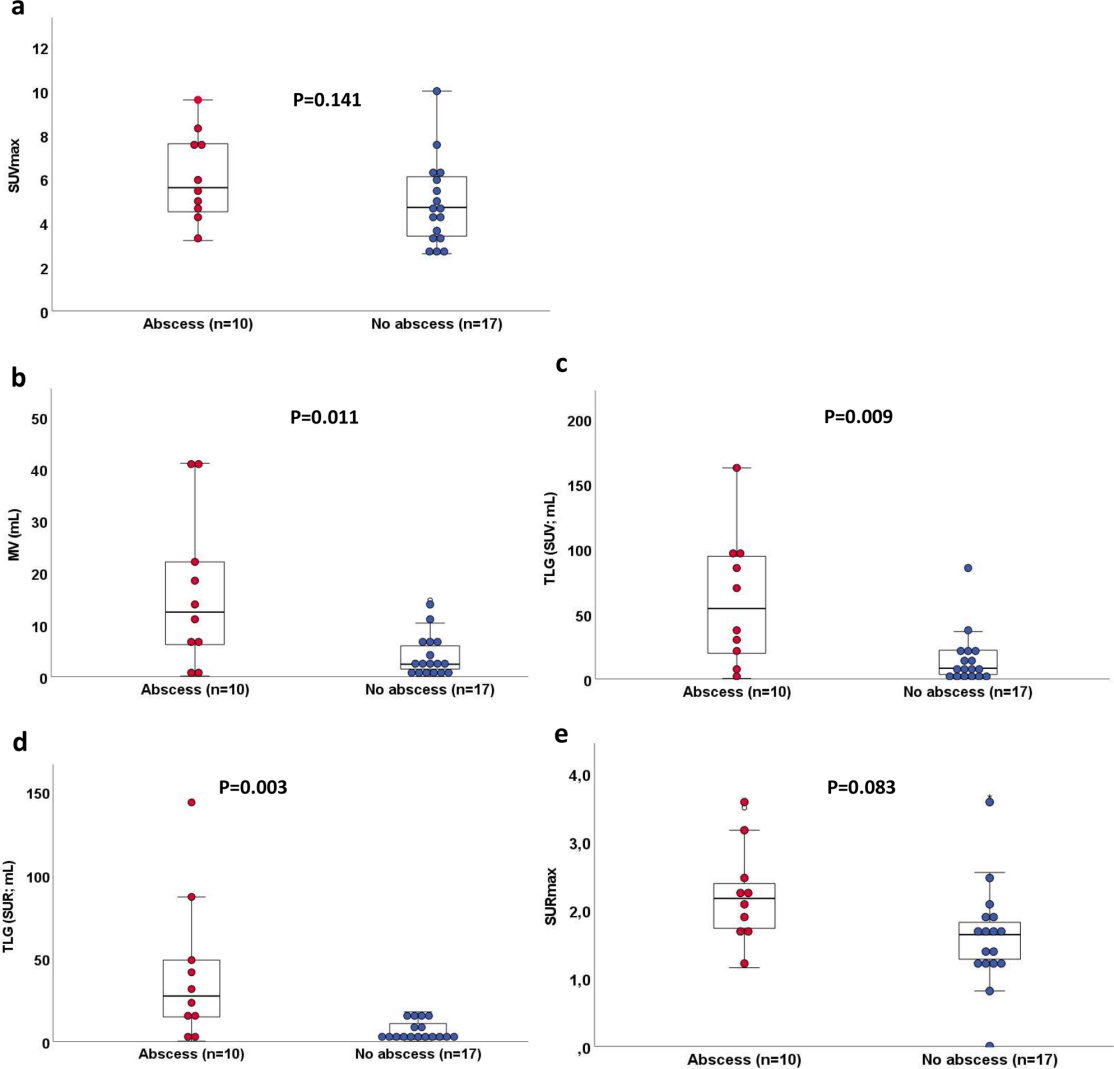
Performance of quantitative ^18^F-FDG PET/CT-derived PET parameters in patients with surgically confirmed IE with and without abscess formation. Box and dot plots of **a** SUVmax, **b** MV, **c** TLG (SUV), **d** TLG (SUR), and **e** SURmax according to presence (in red) or absence of an abscess (in blue) and comparing the median of according values. Box and whisper plots represent the median and the 25, 75, 10, and 90% quantiles, and dots represent individual data points. Significantly higher values for MV, TLG (SUV), and TLG (SUR) were observed in patients with abscess formation as compared to patients without abscess formation. *MV* metabolic volume, *SURmax* standard maximum uptake ratio, *SUVmax* standard maximum uptake value; *TLG* total lesion glycolysis

**Figure 3:**
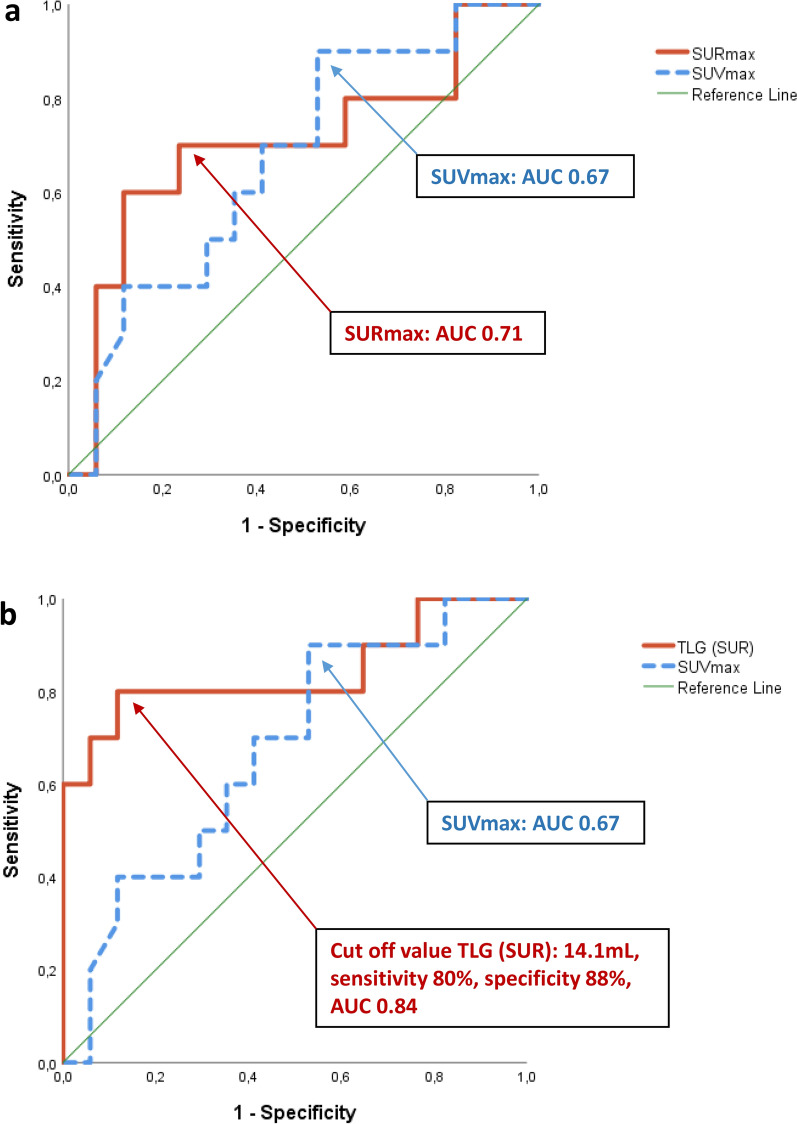
^18^F-FDG PET/CT-derived TLG (SUR) predicts abscess formation in patients with surgically confirmed IE. **a** ROC analysis demonstrates that SURmax as indicated by the red line has a better AUC as compared to SUVmax (dashed blue line) in terms of predicting present abscess formation in patients with surgically confirmed IE. **b** TLG (SUR) as indicated by the red line in the lower panel has a significantly improved AUC as compared to the standard parameter SUVmax (dashed blue line) in terms of predicting abscess formation in patients with surgically confirmed IE. *AUC* area under the curve; *IE* infective endocarditis, *ROC* receiver operating characteristic, *SURmax* standard maximum uptake ratio, *SUVmax* standard maximum uptake value; *TLG* total lesion glycolysis

**Figure 4:**
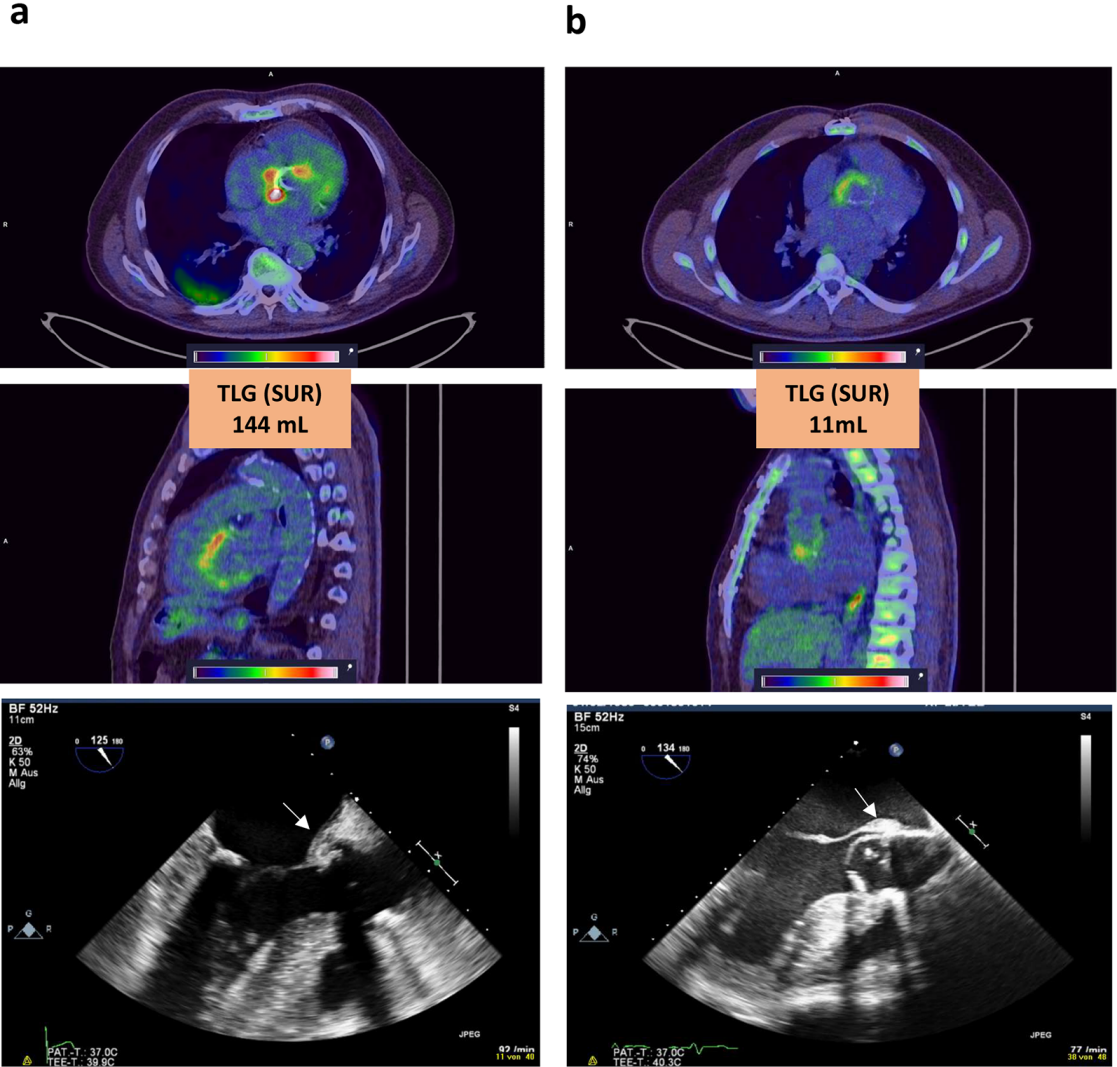
^18^F-FDG PET/CT scans of two patients with surgically confirmed IE. ^18^F-FDG PET/CT scans in the *upper* and *middle panel* demonstrating increased TLG (SUR) in a patient with abscess formation (**a**) as compared to a patient without surgically confirmed abscess formation (**b**). The echocardiographic images are shown in the *lower panel*. The arrow in **a** points at aortic root abscess formation. The arrow in **b** points at a suspicious structure in the area of the aortic root in a patient that had David surgery four years ago that turned out to be no abscess upon surgery. Color scale of PET windowed with SUV 0 to 8. *SUR* standard uptake ratio, *TLG* total lesion glycolysis

^18^F-FDG PET/CT-derived MV (7.4 mL (IQR: 5.9 to 18.5 mL) vs. 1.4 mL (IQR: 0.0 to 2.2 mL)), TLG (SUV) (30.1 mL (IQR: 19.7 to 85.9 mL) vs. 4.8 mL (IQR: 0.1 to 7.9 mL)), and TLG (SUR) (16.6 mL (IQR: 10.7 to 31.4 mL) vs. 1.5 mL (IQR: 0.0 to 3.6 mL)) were increased in patients with true positive as compared to patients with false negative PET scans (data not shown). In patients with PVE (*n* = 17), we observed significantly higher values for MV, TLG (SUV), and TLG (SUR) as compared to patients with non-prosthetic valve IE (NPVE, i.e. patients with NVE or CDRIE *n* = 10), while SUVmax and SURmax did not significantly differ between groups (Figure 1). When dichotomizing into patients with (*n* = 10) and without (*n* = 17) *surgically* confirmed abscess formation, we observed significantly higher values for MV, TLG (SUV), and TLG (SUR) in patients with abscess formation, but not so for SUVmax (*P* = 0.141) and SURmax (*P* = .083, see Figure 2). Importantly, increased TLG (SUR) was still observed when PVE patients were considered individually (*n* = 17, compare Table 5). TLG (SUR) was 41.5 mL (IQR: 14.7 to 86.9 mL) in PVE patients with abscess (*n* = 7) as compared to a TLG (SUR) of only 8.1 mL (IQR: 3.0 to 14.3 mL) in PVE patients without abscess formation (*n* = 10, *P* = .019). In terms of predicting IE-related abscess formation, ROC analyses demonstrated that SURmax had a greater area under the ROC curve (AUC) with a value of 0.706 (CI 0.484 to 0.928) as compared to an AUC of 0.674 (CI 0.465 to 0.882) observed for SUVmax (see Figure 3a). Consequently, TLG (SUR) was identified to have the most favorable area under the ROC curve with a value of 0.841 (CI 0.659 to 1.000) and performed significantly better in predicting abscess formation as compared to the currently used standard PET parameter SUVmax (see Figure 3b for ROC curves and Figure 4 as example of original scans with representative TLG (SUR) values in the presence and absence of an IE-related abscess). In our study cohort, a cut-off value of 14.14 mL for TLG (SUR) resulted in a sensitivity of 80% and a specificity of 88% in predicting IE-related abscess formation. Again, a largely comparable AUC for TLG (SUR) was still observed when PVE patients (AUC was 0.84 (CI 0.60 to 1.08), *n* = 17) and NPVE patients (AUC was 0.86 (CI 0.58 to 1.14) *n* = 10) were considered individually. Interestingly, all patients with abscess formation (*n* = 10) suffered from Staphylococcus-related IE, while there were only nine out of 15 patients in the group without abscess formation that had Staphylococcus subspecies IE (60%, *P* = .022). In that regard, only TLG (SUR) was found to be higher in patients with Staphylococcus subspecies IE when comparing to patients without Staphylococcus subspecies IE (*P* = .036). As blood glucose at the time of ^18^F-FDG injection and macrophage activity act as biological confounders regarding FDG uptake, we also compared blood glucose levels and individual antibiotic regimens in patients with PVE vs. NPVE as well as with and without abscess formation. However, we found no significant differences regarding blood glucose, percentage of patients with ^18^F-FDG PET/CT performed under antibiotic therapy, or duration of antibiogram-based therapy prior to PET/CT scan (data not shown).

**Table 5:** Comparison of SUVmax, SURmax, MV, TLG (SUV), and TLG (SUR) in PVE patients with and without abscess and in NPVE patients with and without abscess

Prosthetic valve IE	Abscess (*n* = 7)	No abscess (*n* = 10)	*P* value
SUVmax	5.4 [4.5–7.6]	4.9 [4.13–6.13]	.536
SURmax	3.0 [2.9–3.9]	2.6 [2.1–2.9]	.055
MV (mL)	13.1 [6.2–40.8]	4.6 [2.1–7.8]	.07
TLG (SUV; mL)	70.0 [19.7–99.0]	17.1 [6.9–26.5]	.088
TLG (SUR; mL)	41.5 [14.7–86.9]	8.1 [3.0–14.3]	.019

Non-prosthetic valve IE	Abscess (*n* = 3)	No abscess (*n* = 7)	*P* value
SUVmax	5.8^a^	3.4 [2.7–6.2]	.183
SURmax	2.1^a^	1.8 [1.4–2.5]	.517
MV (mL)	7.4^a^	0.8 [0.0–2.0]	.117
TLG (SUV; mL)	30.1^a^	3.2 [0.0–8.2]	.067
TLG (SUR; mL)	16.7^a^	1.0 [0.0–4.4]	.117

Values represent the median [interquartile range];

*IE* infective endocarditis, *MV* metabolic volume, *NPVE* non-prosthetic valve endocarditis, *PVE* prosthetic valve endocarditis, *SURmax* standard maximum uptake ratio, *SUVmax* standard maximum uptake value, *TLG* total lesion glycolysis

^a^Interquartile range could not be calculated as *n* < 4

## Discussion

This retrospective analysis is the first to demonstrate that ^18^F-FDG PET/CT-derived TLG (SUR) is capable of predicting endocarditis-related abscess formation in a heterogeneous real-world cohort of patients with surgically confirmed IE.

### ^18^F-FDG PET/CT-derived TLG (SUR) predicts abscess formation in patients with surgically confirmed IE

A major strength of our study cohort is that final IE diagnosis is not simply based on Duke criteria (DC) or final clinical assessment only, but is *surgically proven* in all cases.^15^ Therefore, correlation with ^18^F-FDG PET/CT imaging is reliable. Here, we dichotomized into patients with and without surgically confirmed abscess formation and found that the threshold-guided volumetric parameters MV, TLG (SUV), and TLG (SUR) were significantly increased in patients with abscesses, in which SURmax was increased in trend (*P* = .083). It is important to note that ^18^F-FDG uptake was generally increased paravalvularly or in the myocardium near the heart valves. However, a visual distinction between patients with and without abscess formation was not reliably possible to the human reader. Likewise, visual identification of a rather ^18^F-FDG-free abscess would be beyond the technical capability of a PET/CT scanner with a spatial resolution of only 4 to 6 mm. We therefore aimed to identify IE-related abscess formation (that per se indicates uncontrolled “spreading” inflammation) by the above-mentioned threshold-guided *volumetric* PET parameters. It is important to state that our real-world cohort of patients is heterogeneous and consists of patients with NPVE and PVE which is relevant in so far as patients with PVE had generally higher values of MV, TLG (SUV), and TLG (SUR), which is most likely due to the bigger size of prosthetic valves. However, even when considered individually, TLG (SUR) was still significantly higher in PVE patients with abscesses (by ~fivefold) as compared to their counterparts without abscess formation. In patients with NPVE, TLG (SUR) was numerically increased by ~16-fold, yet not significantly, presumably due to the lower number of patients. Based on these comparable observations, we conclude that combined *metabolic-volumetric* lesion assessment by TLG (SUR) might represent a reliable and valid way to diagnose IE-related abscess by PET. In our heterogeneous real-world cohort, TLG (SUR) was increased by ~sevenfold in patients with abscesses. ROC analyses demonstrated that TLG (SUR) had a favorable AUC with a clinically promising sensitivity of 80% and a specificity of 88% in predicting IE-related abscess formation, which is quite comparable to transesophageal echocardiography (TOE) that is the current gold standard method with a reported sensitivity of 87% and a specificity of 95%.^20^ It needs to be highlighted that quite comparable AUCs of TLG (SUR) were found when PVE and NPVE patients were considered individually, which points to a robust overall performance of TLG (SUR) in predicting abscess formation *independent* of the underlying pathology (i.e. PVE or NPVE).

### Markers of systemic inflammation

In contrast to the good correlation between TLG (SUR) and IE-related abscess formation, we did not find a significant association of any quantitative ^18^F-FDG-derived PET parameter with systemic inflammatory markers such as CRP or leukocytes count in our study population. We interpret this finding in a way that CRP and leukocytes count may rather reflect the severity of the *systemic* inflammatory response as compared to the localized inflammatory activity within the IE affection site in these complexly diseased patients that ultimately required surgical therapy. Furthermore, we found no correlation between MV, TLG (SUV), and TLG (SUR) with vegetation size. As not only size, but also the localization of vegetations, i.e., at valve basis vs. edges, may have an impact on VOI definition and therefore on MV, TLG (SUV), and TLG (SUR), respectively, this could have contributed to this missing correlation. Unfortunately, data on the precise localization of vegetations were available in 10 patients only so that this aspect could not be further investigated here.

### Potential clinical implications

While MV^4,9–14,21,22^ and TLG (SUV) have been extensively studied in the context of oncological disorders, our current study is one of the few that investigates the diagnostic performance of these ^18^F-FDG PET/CT-derived PET parameters (or their SUR-based variant) in the context of IE. In that regard, Avramovic et al have previously compared the diagnostic value of MV with SUVmax in patients with left ventricular assist devices (LVADs).^18^ In this particular setting, they found that inclusion of MV as a metric parameter improved the diagnostic accuracy of ^18^F-FDG PET/CT imaging in detecting LVAD driveline infections, while they also failed to observe a reliable correlation between CRP and leukocytes with MV or SUVmax. However, others did not observe a diagnostic benefit for MV or TLG (SUV) in patients with suspected LVAD infections (mainly due to the wide range of standard deviation as a result of the large range of hypermetabolic volumes and corresponding infection sites^23^).

Our study is the first to demonstrate that IE-related abscess formation may represent a clinical condition in which quantitative lesion assessment by ^18^F-FDG PET/CT-derived TLG (SUR) could be of significant diagnostic benefit. In our distinct study population, IE-associated abscesses were correctly identified in only four out of 10 patients by the current gold standard method TOE. Another three out of 10 patients remained inconclusive following TOE and complementary imaging assessing ^18^F-FDG PET/CT-derived TLG (SUR) as an addition to standard work-up might be particularly helpful in these cases with inconclusive findings. While current ESC guidelines do not encourage PET/CT imaging in NVE patients,^3^ our study shows that assessment of TLG (SUR) is feasible in this group of patients as well. However, ESC guidelines strongly recommend to perform multislice computed tomography (MSCT) first, because it is feasible and practical especially in the critical ill patient and offers similar diagnostic accuracy in detecting IE-related abscesses/pseudoaneurysms as compared to TOE,^3,24^ which might be even improved when performed as ECG-gated MSCT.^25^ In our real-world cohort, no patient underwent ECG-gated MSCT so that we cannot compare ^18^F-FDG-derived TLG (SUR) with MSCT here. Hence, we do not want our data to be interpreted in such a way that PET/CT imaging should be seen as superior to TOE or MSCT, but in a way that *if* PET/CT is performed (and *if* the tight schedule of a busy PET clinic allows for advanced image interpretation), nuclear physicians are strongly encouraged to assess TLG (SUR), because pathological findings may at least result in further multimodal imaging, such as TOE or MSCT, and even surgery eventually. Larger prospective studies are clearly wanted to further investigate the seemingly promising diagnostic performance of quantitative ^18^F-FDG PET/CT-derived TLG (SUR) in the diagnostic work-up of IE-related abscess formation.

## Limitations

Our monocentric retrospective analysis has some important limitations, which have been reported previously.^15^ An important limitation of our study is the rather small number of patients that we could include here. Due to the retrospective nature of this study, indication for ^18^F-FDG PET/CT was not based on current ESC guidelines only (i.e. for PVE patients > 3 months after valve implantation only), but included patients < 3 months after surgery and patients with definite IE diagnosis according to traditional DC as well, which could lead to an important selection bias. Suppression of myocardial nuclide uptake was not routinely performed, since patients were mostly referred to the Department of Nuclear Medicine for the detection of an infectious focus in general. The according limitations in patient preparation and image acquisition may have likely contributed to the substantially lower sensitivity of ^18^F-FDG PET/CT in our study population as compared to previous studies,^26^ and might have impeded the endocarditis and abscess diagnosis as well. Finally, there were *no* patients with true negative scans included in this cohort of patients that all had surgically confirmed IE so that ROC-based analyses regarding the diagnostic accuracy of quantitative IE assessment modalities are not possible here.

## Conclusion

We conclude that ^18^F-FDG PET/CT-derived assessment of TLG (SUR) may provide a novel diagnostic tool in the diagnostic work-up of endocarditis-related abscess formation that merits further prospective investigation.

## New knowledge gained

In this retrospective analysis of patients with surgically confirmed IE, TLG (SUR) calculated as the product of MV × SURmean predicted the presence of endocarditis-related abscesses with a sensitivity of 80% and a specificity of 88%. Therefore, combining the advantages of metabolic lesion assessment by SUR and volumetric lesion assessment using MV might result in a better diagnostic accuracy of ^18^F-FDG PET/CT and may translate into a more individualized therapeutic approach. A potential field of application could be TLG (SUR)-driven identification of patients with IE-associated abscess that should be referred to subsequent multimodal imaging (i.e. TOE or MSCT) and surgery eventually.

### Supplementary Information

Below is the link to the electronic supplementary material.

Supplementary file1 (MP3 6520 kb)

Supplementary file2 (PPTX 13728 kb)

## Abbreviations

CDRIE: Cardiac device-related infective endocarditis
IE: Infective endocarditis
MV: Metabolic volume
NVE: Native valve endocarditis
NPVE: Non-prosthetic valve endocarditis (i.e. patients suffering from CDRIE or NVE)
PVE: Prosthetic valve endocarditis
SUR: Standard uptake ratio
SUV: Standard uptake value
TLG: Total lesion glycolysis
VOI: Volume of interest
