# Supplementary material for: 18F-FDG PET/CT-derived total lesion glycolysis predicts abscess formation in patients with surgically confirmed infective endocarditis: Results of a retrospective study at a tertiary center
Source: J Nucl Cardiol. 2023 Jun 1;30(6):2400–14. doi: 10.1007/s12350-023-03285-5 (PMC10682046; doi:10.1007/s12350-023-03285-5)
Supplement: Supplementary file 2 — Supplementary file2 (PPTX 13728 kb) [file 12350_2023_3285_MOESM2_ESM.pptx]

## Slide 1
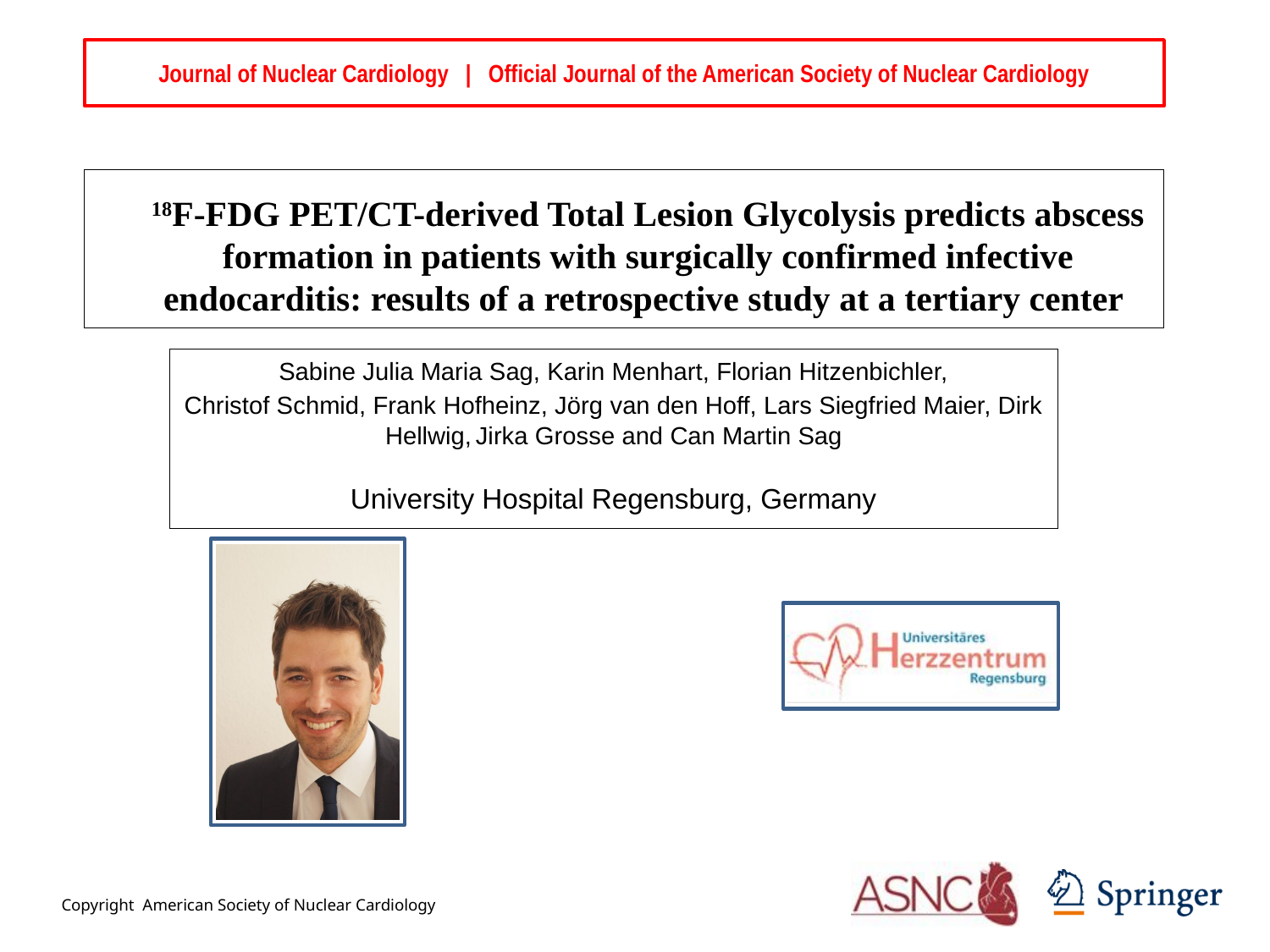

Journal of Nuclear Cardiology | Official Journal of the American Society of Nuclear Cardiology
# 18F-FDG PET/CT-derived Total Lesion Glycolysis predicts abscess formation in patients with surgically confirmed infective endocarditis: results of a retrospective study at a tertiary center
Sabine Julia Maria Sag, Karin Menhart, Florian Hitzenbichler,
Christof Schmid, Frank Hofheinz, Jörg van den Hoff, Lars Siegfried Maier, Dirk Hellwig, Jirka Grosse and Can Martin Sag
University Hospital Regensburg, Germany
Copyright American Society of Nuclear Cardiology

## Slide 2
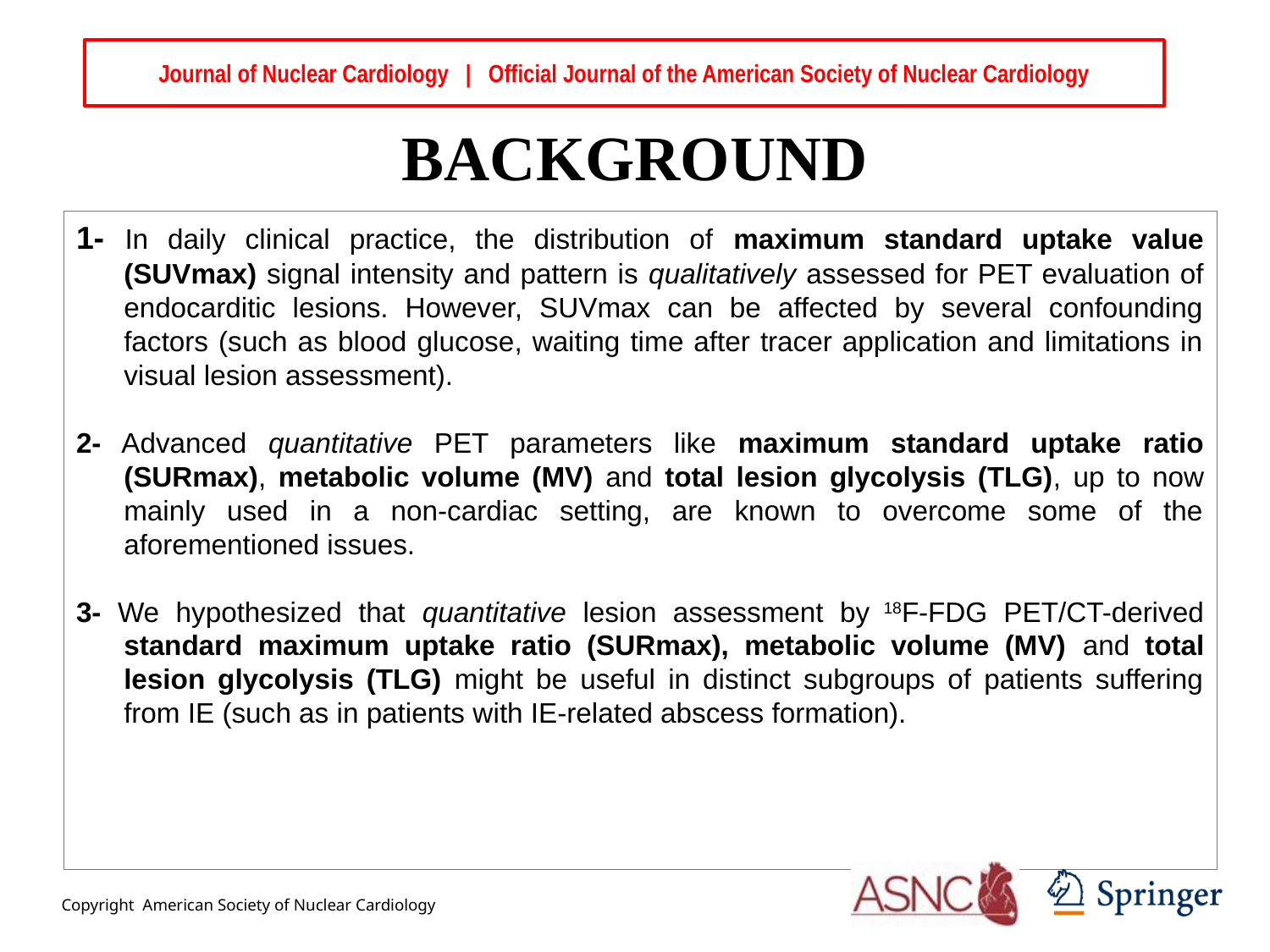

Journal of Nuclear Cardiology | Official Journal of the American Society of Nuclear Cardiology
# BACKGROUND
1- In daily clinical practice, the distribution of maximum standard uptake value (SUVmax) signal intensity and pattern is qualitatively assessed for PET evaluation of endocarditic lesions. However, SUVmax can be affected by several confounding factors (such as blood glucose, waiting time after tracer application and limitations in visual lesion assessment).
2- Advanced quantitative PET parameters like maximum standard uptake ratio (SURmax), metabolic volume (MV) and total lesion glycolysis (TLG), up to now mainly used in a non-cardiac setting, are known to overcome some of the aforementioned issues.
3- We hypothesized that quantitative lesion assessment by 18F-FDG PET/CT-derived standard maximum uptake ratio (SURmax), metabolic volume (MV) and total lesion glycolysis (TLG) might be useful in distinct subgroups of patients suffering from IE (such as in patients with IE-related abscess formation).
Copyright American Society of Nuclear Cardiology

## Slide 3
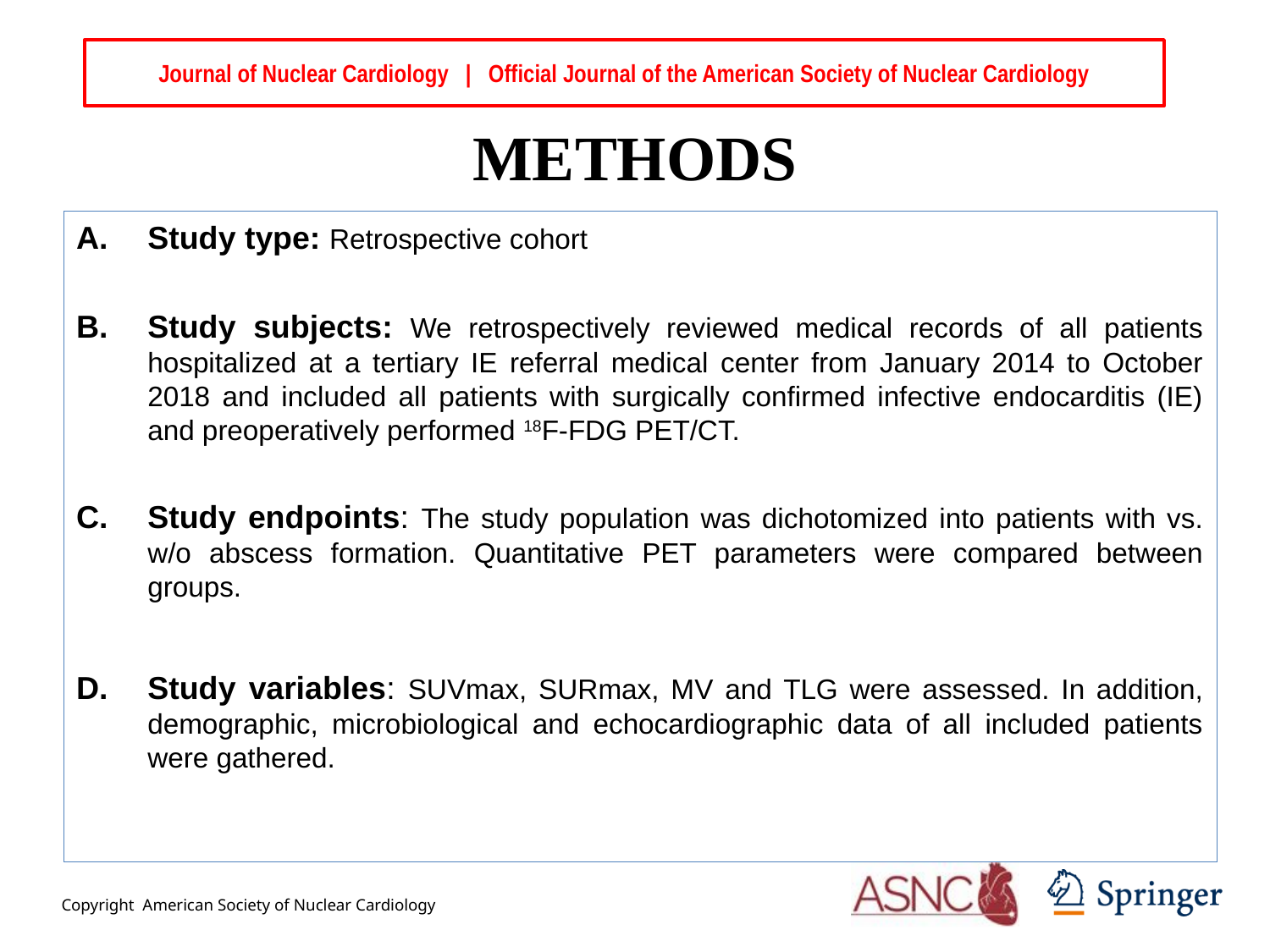

Journal of Nuclear Cardiology | Official Journal of the American Society of Nuclear Cardiology
# METHODS
Study type: Retrospective cohort
Study subjects: We retrospectively reviewed medical records of all patients hospitalized at a tertiary IE referral medical center from January 2014 to October 2018 and included all patients with surgically confirmed infective endocarditis (IE) and preoperatively performed 18F-FDG PET/CT.
Study endpoints: The study population was dichotomized into patients with vs. w/o abscess formation. Quantitative PET parameters were compared between groups.
Study variables: SUVmax, SURmax, MV and TLG were assessed. In addition, demographic, microbiological and echocardiographic data of all included patients were gathered.
Copyright American Society of Nuclear Cardiology

## Slide 4
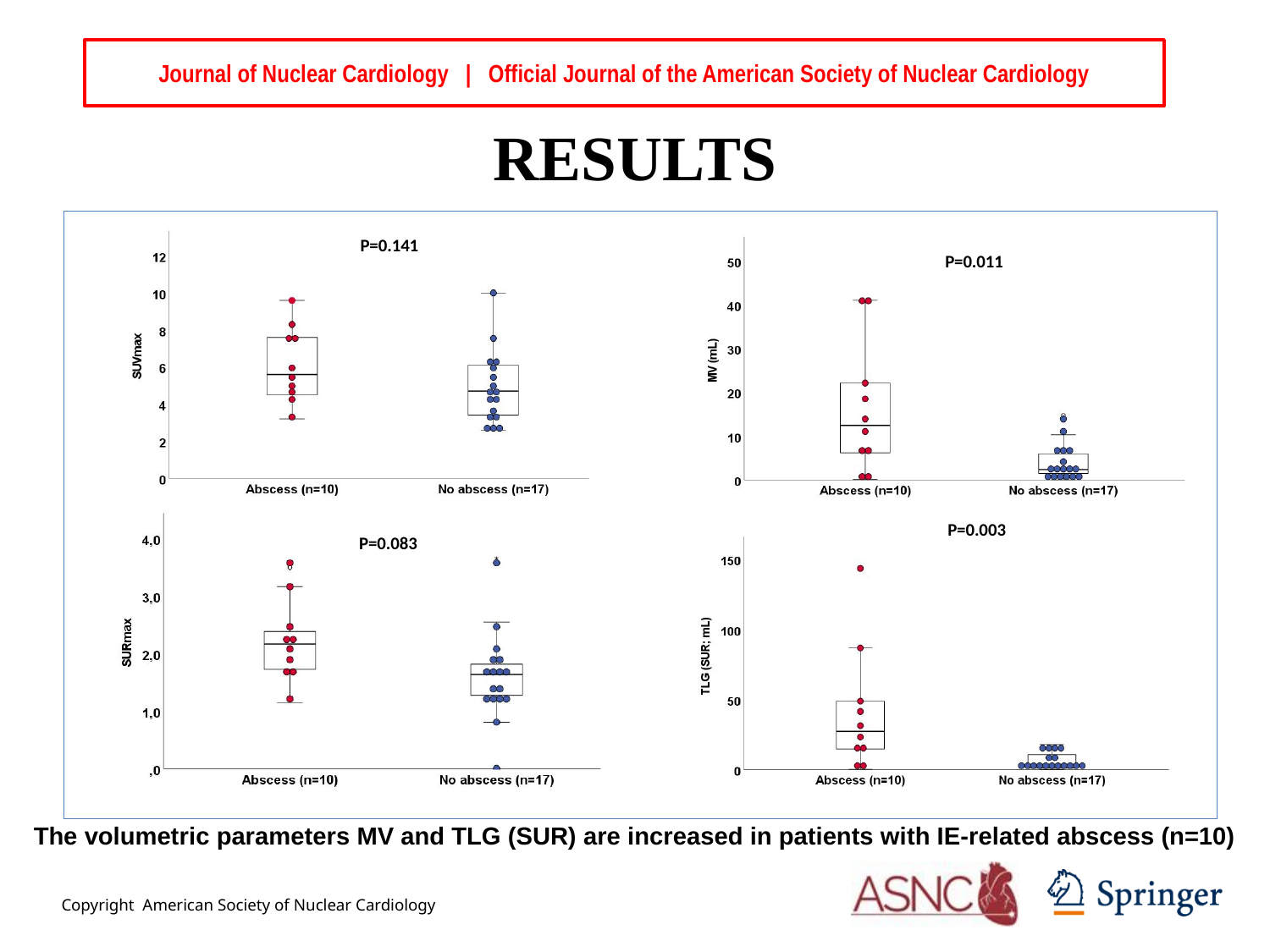

Journal of Nuclear Cardiology | Official Journal of the American Society of Nuclear Cardiology
# RESULTS
P=0.141
P=0.011
P=0.003
P=0.083
The volumetric parameters MV and TLG (SUR) are increased in patients with IE-related abscess (n=10)
Copyright American Society of Nuclear Cardiology

## Slide 5
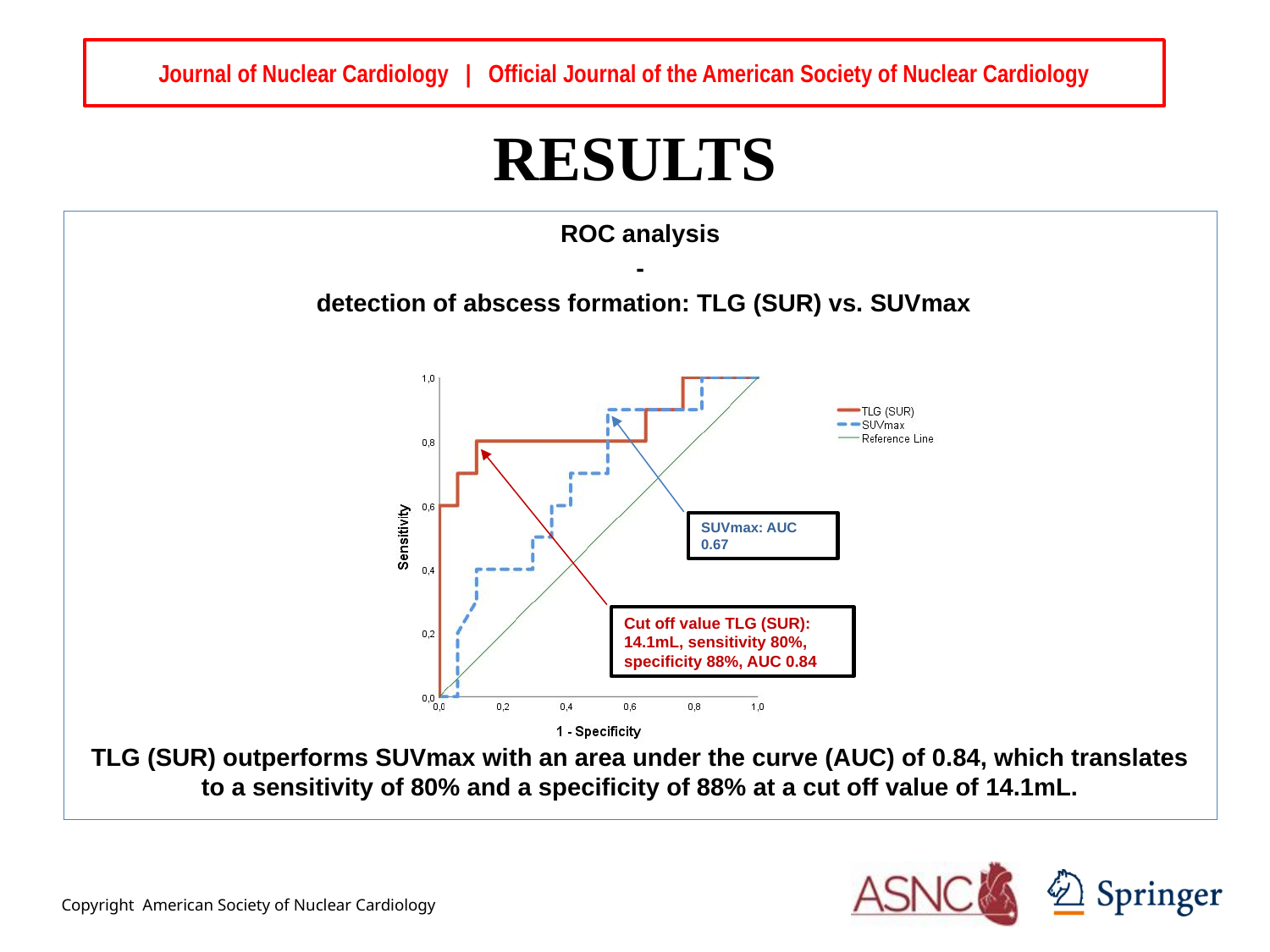

Journal of Nuclear Cardiology | Official Journal of the American Society of Nuclear Cardiology
# RESULTS
ROC analysis
-
 detection of abscess formation: TLG (SUR) vs. SUVmax
TLG (SUR) outperforms SUVmax with an area under the curve (AUC) of 0.84, which translates to a sensitivity of 80% and a specificity of 88% at a cut off value of 14.1mL.
SUVmax: AUC 0.67
Cut off value TLG (SUR): 14.1mL, sensitivity 80%, specificity 88%, AUC 0.84
Copyright American Society of Nuclear Cardiology

## Slide 6
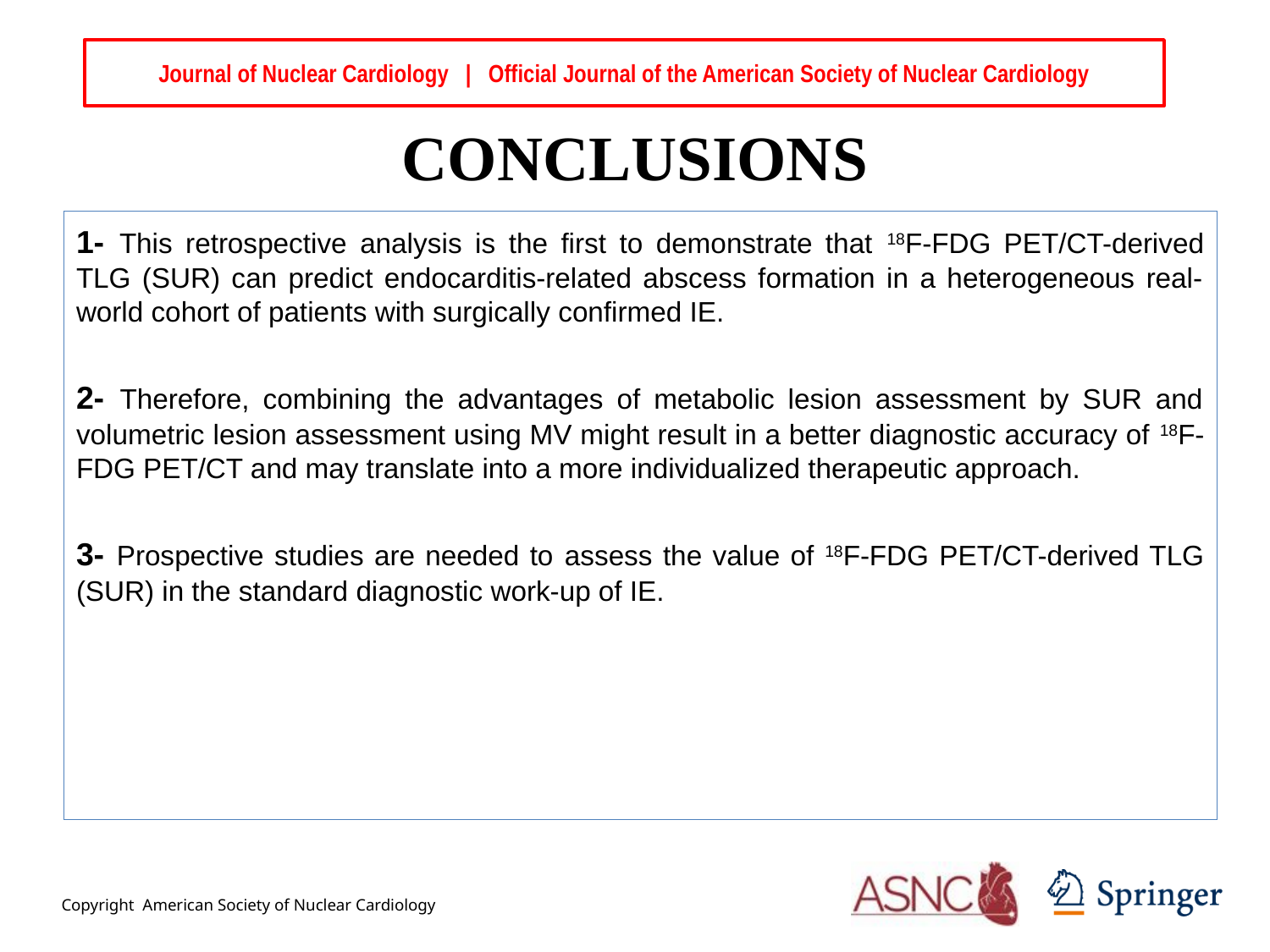

Journal of Nuclear Cardiology | Official Journal of the American Society of Nuclear Cardiology
# CONCLUSIONS
1- This retrospective analysis is the first to demonstrate that 18F-FDG PET/CT-derived TLG (SUR) can predict endocarditis-related abscess formation in a heterogeneous real-world cohort of patients with surgically confirmed IE.
2- Therefore, combining the advantages of metabolic lesion assessment by SUR and volumetric lesion assessment using MV might result in a better diagnostic accuracy of 18F-FDG PET/CT and may translate into a more individualized therapeutic approach.
3- Prospective studies are needed to assess the value of 18F-FDG PET/CT-derived TLG (SUR) in the standard diagnostic work-up of IE.
Copyright American Society of Nuclear Cardiology
